# Early Implementation of THAM for ICP Control: Therapeutic Hypothermia Avoidance and Reduction in Hypertonics/Hyperosmotics

**DOI:** 10.1155/2014/139342

**Published:** 2014-12-04

**Authors:** F. A. Zeiler, L. M. Gillman, J. Teitelbaum, M. West

**Affiliations:** ^1^Section of Neurosurgery, Department of Surgery, University of Manitoba, Winnipeg, MB, Canada R3A 1R9; ^2^Section of Critical Care Medicine, Department of Medicine, University of Manitoba, Winnipeg, MB, Canada R3A 1R9; ^3^Section of General Surgery, Department of Surgery, University of Manitoba, Winnipeg, MB, Canada R3A 1R9; ^4^Section of Neurocritical Care, Montreal Neurological Institute, McGill University, Montreal, QC, Canada H3A 2B4; ^5^Section of Neurology, Montreal Neurological Institute, McGill University, Montreal, QC, Canada H3A 2B4

## Abstract

*Background*. Tromethamine (THAM) has been demonstrated to reduce intracranial pressure (ICP). Early consideration for THAM may reduce the need for other measures for ICP control.* Objective*. To describe 4 cases of early THAM therapy for ICP control and highlight the potential to avoid TH and paralytics and achieve reduction in sedation and hypertonic/hyperosmotic agent requirements.* Methods*. We reviewed the charts of 4 patients treated with early THAM for ICP control.* Results*. We identified 2 patients with aneurysmal subarachnoid hemorrhage (SAH) and 2 with traumatic brain injury (TBI) receiving early THAM for ICP control. The mean time to initiation of THAM therapy was 1.8 days, with a mean duration of 5.3 days. In all patients, after 6 to 12 hours of THAM administration, ICP stability was achieved, with reduction in requirements for hypertonic saline and hyperosmotic agents. There was a relative reduction in mean hourly hypertonic saline requirements of 89.1%, 96.1%, 82.4%, and 97.0% for cases 1, 2, 3, and 4, respectively, comparing pre- to post-THAM administration. Mannitol, therapeutic hypothermia, and paralytics were avoided in all patients.* Conclusions*. Early administration of THAM for ICP control could potentially lead to the avoidance of other ICP directed therapies. Prospective studies of early THAM administration are warranted.

## 1. Introduction

Tromethamine (THAM) is a non-CO_2_ generating buffer solution that has been utilized for a variety of clinical applications [1], including the control of intracranial pressure (ICP) [2–4]. Cerebral lactic acidosis after injury has been linked to edema formation and is postulated to be a major contributor to elevated intracranial pressures [5]. Attenuation of such acidosis via non-CO_2_ buffer compounds, such as THAM, can allow stability in ICP and an overall reduction in pressure.

Both human and animal studies [6] exist demonstrating the ICP reduction effects of THAM [7]. Recent literature review of the human literature demonstrates an Oxford 2b, GRADE B level of evidence that THAM reduces ICP in traumatic brain injury (TBI) and stroke populations, with minimal adverse effects [8].

Other means of ICP reduction include therapeutic hypothermia (TH), involving external or intravascular cooling of the patient [9, 10]. Though effective in reduction of ICP, this method carries significant morbidity and complications [10]. Thus, if possible, avoidance of TH in ICP control is desired. Early implementation of THAM in patients with ICP issues may provide a treatment that can avoid TH and its inherent complications.

Within we describe 4 cases of patients with elevated ICP in which early administration of THAM led to avoidance of TH and paralytics and a reduction in sedation and hypertonic/hyperosmotic agent usage.

## 2. Methods

We retrospectively reviewed the charts of 4 patients who received early implementation of THAM therapy for elevated ICP. Data on patient demographics, admission diagnosis, THAM treatment characteristics, pre- and post-THAM hypertonic and hyperosmotic solution requirements, sedation requirements, and patient outcomes were recorded.

## 3. Results

### 3.1. THAM Treatment Characteristics

The THAM treatment protocol for all patients was the same. A 0.3 molar solution of THAM was administered, with a 2 mL/kg bolus over 1 hour, followed by a continuous infusion of 1 mL/kg/hr. The mean time to administration of THAM was 1.8 days after injury (range: 1–3 days). The mean duration of THAM therapy was 5.3 days (range: 3–7 days).

### 3.2. THAM Response

#### 3.2.1. Case 1

A 62-year-old male was admitted after assault with a large right occipital hyperacute epidural hematoma. His initial Glasgow Coma Score (GCS) was 4T. He was emergently taken to the operative room for evacuation of the hematoma. An ICP monitor was placed intraoperatively.

During the first 12 hours postoperatively, his ICP became difficult to control ranging from 15 to 35 mm Hg, with multiple sustained elevations above 20 mm Hg. The total volume of hypertonic saline administered during this time was 840 mL, with the mean hourly 7.3% saline requirements at 44.2 mL/hr. The total amount of mannitol administered was 100 gm. The sedative requirements at this time were as follows: fentanyl 400 mcg/hr, midazolam 20 mg/hr, and propofol 5 mg/kg/hr.

We initiated THAM therapy 19 hours after his operation. Within 6 hours of initiation of THAM treatment, the fluctuations in his ICP above 20 mm Hg reduced dramatically, with 7.3% hypertonic saline administered only once (total volume of 140 mL) during the remainder of his hospital stay. The mean hourly 7.3% saline requirements after THAM were 4.8 mL/hr. No mannitol was required after THAM administration. Therapeutic hypothermia and paralytics were completely avoided. Sedative dosing remained unchanged. No complication related to THAM was identified during the 3 days of therapy.

Unfortunately, given the poor prognosis and magnetic resonance imaging evidence of brainstem and bilateral hemispheric watershed infarcts, it was elected by the family to withdrawal care.

#### 3.2.2. Case 2

A 48-year-old male was admitted after falling down the stairs while intoxicated. His initial GCS on presentation was 5T, and CT of the head demonstrated small right occipital epidural hematoma secondary to a skull fracture over the transverse sinus. Also, there were significant bifrontal lobe contusions. He was admitted to the ICU, and an ICP monitor was placed.

The patient's initial ICP was 24 mm Hg, and prior to THAM therapy it ranged from 15 to 27 mm Hg. On postinjury day number 1, fentanyl was titrated to 150 mcg/hr, midazolam to 15 mg/hr, and propofol to 5 mg/kg/hr. A total of 280 mL of 7.3% hypertonic saline was utilized to control sustained ICP fluctuations above 20. On postinjury day number 2, ICP continued to climb requiring fentanyl at 350 mcg/hr, midazolam at 30 mg/hr, and propofol at 5 mg/kg/hr. The total volume of hypertonic saline utilized in this 24-hour period was 280 mL. On postinjury day number 3, propofol was discontinued due to increasing lactate and was replaced with ketamine at 35 mcg/kg/min. The ICP was increasingly hard to control throughout the day, requiring a total of 560 mL of hypertonic saline to control sustained fluctuations above 20 mm Hg. Over these 72 hours the patient averaged a 15.5 mL/hr of 7.3% hypertonic saline requirement. Thus, THAM therapy was initiated in attempt to avoid hypothermia and paralytics.

Within 12 hours of initiating THAM treatment, ICP fluctuations above 20 mm Hg reduced dramatically. The ICP post-THAM remained between 9 and 20 mm Hg. Over the following 72 hours of THAM therapy, a total of 420 mL of hypertonic saline was utilized (140 mL/day), for a mean 0.6 mL/hr requirement over this period. No mannitol was required post-THAM administration. Therapeutic hypothermia was completely avoided.

The patient was discontinued from THAM therapy after 4 days, was extubated, and eventually transferred to rehabilitation with only minor cognitive deficits. No complication related to THAM was identified.

#### 3.2.3. Case 3

A 56-year-old female was admitted with a Fisher grade 4, Hunt and Hess grade 4 subarachnoid hemorrhage (SAH) secondary to an anterior communicating artery aneurysm. Initial GCS was 5T. After external ventricular drain (EVD) insertion, her GCS improved to 7T, with an opening pressure of 32. The EVD was venting 5–10 mL/hr, open at 20 cm above the tragus, for the duration of her stay in the intensive care unit (ICU). Tranexamic acid was started and continued for the first 72 hours of admission.

Over the first 18 hours of admission her ICP was managed as follows. Aggressive treatment of ICP was conducted utilizing the following sedation: fentanyl titrated to 300 mcg/hr, midazolam titrated to 20 mg/hr, and propofol titrated to 5 mg/kg/hr. Hypertonic saline (7.3%) was administered 3 times for ICP spikes above 20 mmHg, for a total volume of 420 mL. Her mean hourly hypertonic saline requirement was 26.7 mL/hr. Mannitol was given twice for a total of 100 gm, leaving serum osmolarity at 315.

At this point the patient was unable to tolerate lying flat for angiographic management of her ruptured aneurysm. Tromethamine was initiated to avoid hypothermia and paralytics. On postbleed day number 2, her hypertonic saline requirements stayed at 320 mL over the 24-hour period, but a significant reduction in ICP fluctuations above 20 was noted after 12 hours of THAM therapy, allowing a reduction of propofol to 3 mg/kg/hr and complete avoidance of mannitol. On postbleed day number 3, propofol was completely turned off, with ongoing avoidance of mannitol usage. The hypertonic saline requirement over the 24 hours was 140 mL. On postbleed day number 4, mannitol and propofol were avoided, and midazolam was reduced to 20 mg/kg. The total hypertonic requirement was 280 mL over the 24 hours. No further hypertonic saline was needed for postbleed days number 5 to 7. Her mean hourly hypertonic saline requirement after initiation of THAM was 4.7 mL/hr. No complication related to THAM was identified.

On postbleed day number 7, the patient had an acute spike in her ICP to 74, with bilateral blown pupils. Repeat computed tomography (CT) of the brain demonstrated a rebleed from the aneurysm. Sedation and treatment were withdrawn and the patient died. The THAM therapy was administered for a total of 7 days.

#### 3.2.4. Case 4

A 43-year-old male was admitted on postbleed day number 4 with a Fisher grade 4, Hunt and Hess grade 3 SAH secondary to a left posterior communicating artery aneurysm. He presented clinical vasospasm of the left middle cerebral artery territory, with right hemineglect and dysphasia. Emergent microsurgical clipping of his aneurysm was completed with 6 hours of admission, and he was transferred to ICU postoperatively for management of his vasospasm. He had an ICP monitor placed intraoperatively.

He failed hypertensive therapy with mean arterial blood pressure (MABP) of 140 to 150 mm Hg, with no clinical improvement. Continuous intravenous milrinone therapy was initiated with complete resolution of his symptoms and MABP of 90 to 100 mm Hg.

The ICP was fluctuating from 18 to 31 mm Hg during the first 8 hours postoperatively. He received multiple doses of 7.3% hypertonic saline for a total of 700 mL, increasing his serum sodium to 154. The mean hourly hypertonic saline requirement was 87.5 mL/hr during this period prior to THAM. His serum osmolarity at that time was 315. Given the concerns overclouding his clinical exam with sedation during active treatment of symptomatic cerebral vasospasm post-SAH, we elected trial THAM therapy.

Within 6 hours of THAM administration the fluctuations in ICP above 20 were dramatically reduced with ICP ranging from 11 to 23 mm Hg. The requirements for hypertonic saline were reduced to 420 mL over the following 24 hours, after which no hypertonic saline was needed for ICP control during the remaining ICU course. Mannitol usage was avoided completely during THAM treatment. The THAM therapy was continued for a total of 7 days. The patient's mean hourly hypertonic saline requirement after initiation of THAM was 2.6 mL/hr. No complication related to THAM was identified.

At no point was sedation required to control ICP in this patient, thus preserving the neurological examination. On postbleed day number 7, the patient developed worsening focal deficits secondary to vasospasm, requiring angioplasty. These deficits would have likely been missed if on sedatives for ICP control at that time.

The patient eventually recovered from his deficits with mild expressive dysphasia and was transferred to stroke rehabilitation.

## 4. Discussion

The management of refractory elevated ICP is challenging. Despite maximal management with optimal patient positioning, control of pCO_2_ hypertonic/hyperosmotic agents, intravenous sedatives, paralytics, and cerebrospinal fluid (CSF) diversion, ICP control can be difficult.

The use of TH in traumatic brain injury has been extensively investigated as a means of ICP control and neuroprotection [9, 10]. Though data on the impact of TH on patient outcome is unclear, the therapy is quite effective at reduction of increased ICP [9]. The caveat to TH is the complications associated with it implementation. Coagulopathy, hypotension, cardiac dysfunction, and infections complicate TH in all of its applications [10].

Tromethamine provides a non-CO_2_ buffering of cerebral lactic acidosis in the setting of brain injury, leading to a reduction in edema formation and ICP reduction [1]. Though not standard front line therapy for elevated ICP, THAM carries the potential to stabilize ICP fluctuations and reduce the need for more aggressive measures, including paralytics and TH.

We reviewed 4 cases of early implementation of THAM for ICP control in 2 TBI and 2 SAH patients. Tromethamine was initiated at a mean of 1.8 days after injury, for a mean duration of 5.3 days. With this small experience we were able to avoid TH in all patients. Similarly, paralytic agents were avoided. After 6 to 12 hours of THAM infusion, significant reductions in the number of sustained ICP fluctuations above 20 mm Hg occurred in all patients. A dramatic reduction in the requirement of hypertonic saline was noted after the initiation of THAM in all patients. The relative reduction in the mean hourly hypertonic saline requirements pre-THAM compared to post-THAM was 89.1%, 96.1%, 82.4%, and 97.0% for cases 1, 2, 3, and 4, respectively. We were able to discontinue propofol usage in case 3, and completely avoid sedation in one patient (case 4) that was suffering from symptomatic cerebral vasospasm. No complications occurred related to THAM therapy. Two patients succumbed to their underlying neuropathology.

We believe this case series displays a few important and interesting findings. First, early implementation of THAM for ICP control is feasible. Second, a dramatic reduction in mean hourly hypertonic saline requirements was displayed in these 4 patients once THAM was initiated. However, this may not necessarily be the response in all patients, and our series only represents a select group. Third, a significant reduction in sustained ICP fluctuations above 20 mm Hg was noted. Fourth, TH and paralytic agents were avoided in all patients. However, given the small patient cohort, this is difficult to interpret. Fifth, sedation reduction was displayed in one SAH patient (case 3). Similarly, sedation avoidance was achieved in case 4, allowing preservation of the neurological examination during symptomatic post-SAH vasospasm. This afforded us the opportunity to determine deterioration of his neurological status leading to angioplasty therapy. Sixth, mannitol usage was avoided in all patients after THAM administration. Finally, no complications related to THAM therapy were identified.

Despite the interesting findings in this series, significant limitations to our study exist. First, the retrospective nature limits our ability to generalize our findings to all patients with elevated ICP. Second, we only have 4 patients in our series; thus our findings are based on a new institutional experience with early THAM for ICP control and may not reflect the experience in a larger series. Third, outcomes related to the underlying neuropathology did not reflect the positive results obtained in the other areas of these patients medical management of their ICP issues. Finally, there exists a significant potential for publication bias with our series given the positive results encountered.

Even though the limitations exist, as outlined, we still believe this study displays the potential that early THAM administration carries in the setting of ICP control. The trend towards reduction in hypertonic/hyperosmotic agent dosing, mannitol, and sedation and avoidance of TH and paralytics warrant further prospective evaluation of early THAM for ICP control in order to determine its effect on these factors. Similarly, the effect of early THAM therapy on patient outcome has yet to be determined.

## 5. Conclusions

Early THAM administration in the setting of increased ICP carries the potential for avoidance of TH and paralytics and a reduction in the volume of hypertonic/hyperosmotic agents and sedation requirements. Further prospective evaluation of early THAM for ICP control needs to occur.
